# Surface Versus Penetrative rTMS Stimulation May Be More Effective for AD Patients with Cerebrovascular Disease

**DOI:** 10.1177/26331055251328355

**Published:** 2025-03-25

**Authors:** Brian J Lithgow, Chandan Saha, Zeinab Dastgheib, Zahra Moussavi

**Affiliations:** 1 Biomedical Engineering, University of Manitoba, Winnipeg, Canada; 2 Monash Alfred Psychiatry Research Centre, Melbourne, Australia; 3 Riverview Health Centre, Winnipeg, Manitoba, Canada

**Keywords:** rTMS, Alzheimer’s Disease, cerebrovascular disease, ADAScog

## Abstract

Repetitive Transcranial Magnetic Stimulation (rTMS) has been applied as an investigational therapy for Alzheimer’s Disease (AD). The recent largest (N = 135) double-blind study with 6 months post-treatment follow-up investigating rTMS efficacy as a treatment for AD found about 72% of participants in each group of active and sham were positively responsive to rTMS (using Magstim AirFilm active and sham coils). Since the used sham coil produced about 25.3% of the peak active stimulus, it was hypothesized it could evoke a measurable response in AD patients. This study looks at the details of the above study’s sham responses to determine why and how such a response might occur and how cerebrovascular symptomatology may have impacted that response. In the above-mentioned study, 90 and 45 patients were randomly assigned to active and sham groups, respectively. Those with modified Hachinski Ischemic Scores (HIS) below and above 2 were labeled AD_2_ and ADcvd_2_, respectively. Analysis of the primary outcome measure ADAS-Cog score change from baseline to post-treatment and follow-ups showed the ADcvd_2_ in the sham group had a significantly (*p* = .034) greater improvement or less decline at post-treatment and follow-up sessions compared to the ADcvd_2_ in the active group. Additionally, the improvement of the ADcvd_2_ sham compared to those in the active group persisted longer. Also, there was a significant (*p* = .036) improvement for AD_2_ individuals in the active compared to AD_2_ sham stimulation group at 2-months post-treatment. Overall, the sham rTMS stimulus did evoke a measurable response which was more effective for ADcvd_2_ in sham than ADcvd_2_ in active support of a vascular mechanism likely linked to the shallower sham stimulus penetration.

## Introduction

Transcranial Magnetic Stimulation (TMS) is a procedure, in which a current passes through a coil placed on the scalp producing a magnetic field.^1^ There are several different coil designs for TMS, out of which the Figure-8 design is the most common. The magnetic field of an active Figure-8 coil is concentrated over its center and when given at an individual’s resting motor threshold, passes through the skull to the brain, wherein a small current is induced that excites neurons. In contrast, the magnetic field of the sham figure-8 coil is scattered and while its intensity reaches up to 25.3% of the active coil^2,3^ the induced current is not strong enough for neuronal excitation.

In the last decade, many groups have applied repetitive TMS (rTMS) at high frequencies (eg, 20 Hz) as a treatment for Alzheimer’s disease (AD).^4-10^ Most were pilot studies applying rTMS in small samples. They mostly applied the pulses to the dorsolateral prefrontal cortex (DLPFC) either only to the left side^4-6^ or bilaterally^7,9,10^ generally with a high-frequency (10-20 Hz). Others applied stimulation to the Broca and Wernicke areas,^7^ or the precuneus.^8^ The largest study (N = 135) to date^11^ with 6 months post-treatment follow-up investigating rTMS efficacy as a treatment for AD has found about 72% of participants in each group of active and sham were positively responsive to rTMS (using Magstim AirFilm active and sham coils). The authors hypothesized the used sham coil, which produces about 25.3% of the active coil peak stimulus,^2,3^ was evoking a measurable response in participants. Also, as the level of cerebrovascular disease (cvd) may be an impacting factor on rTMS efficacy, as implied by a recent study.^12^ This study will examine the details of that study’s^11^ cvd active and cvd sham groups’ responses to determine why and how such a response may occur by group considering cerebrovascular symptomatology.

The above-mentioned study^11^ was a multisite double-blind randomized clinical trial investigating the efficacy of rTMS as a treatment for individuals with mild to moderate AD. Doses of rTMS were either 2 or 4 weeks of treatment (5 days/week) with pulses given at 20 Hz in 1.5 second trains with 10-second intertrain intervals for a total of 750 pulses applied serially to the left and right DLPFC; the exact location of the target was determined using BrainSite Neuro-navigation system using each participant’s MRI brain scan. The protocol was the same for the sham stimulation applied over 4 weeks. The primary outcome measure was the change of Alzheimer’s Disease Assessment Scale-Cognitive Subscale (ADAS-Cog) score with respect to baseline. The results showed both short and long-term benefits of active rTMS treatment but also showed similar benefits for the sham coil treatment.

Compared to the active coil, the sham coil produced a much shallower penetration but a much wider surface stimulation area (supplementary data of Moussavi et al.^11^). The residual brain stimulation is a limitation of the sham coil in order to provide the same sensory experience as the active coil.^13^ The active Figure-8 TMS coil creates magnetic fields in each of the coil loops in the opposite direction; comparatively, the sham coil creates a magnetic field similar to a single large oval loop by having the magnetic field in the same direction for both loops.^14^ The result is the perpendicular magnetic field strength further away from the coil (eg, 20 cm) is stronger for the sham coil compared to the active coil.^2,3^ These significant but weaker fields from the sham TMS coil can modify the cortical excitability of the neurons.^3,11^ Surface (sham) rather than penetrative (active) stimuli likely encompass a different mode of stimulation and neural plasticity. We hypothesize that the sham rTMS stimulus does evoke a measurable response which is more effective on those with cerebrovascular symptomatology/disease (ADcvd) which is supportive of a vascular mechanism likely linked to the shallower sham stimulus penetration. In other words, that the level of cerebrovascular disease may be an impacting factor on rTMS efficacy, as implied by a recent study.^12^ If so a re-examination of the rTMS efficacy within 2 months of treatment (given nearly all improvement was observed in this period^11^) without the ADcvd population would likely be illuminating.

The current study tests 2 main separate hypotheses: (1) AD with cerebrovascular symptomatology/disease (ADcvd) groups have significantly different responses across time when receiving either active or sham stimuli and (2) Within 2 months of treatment AD with no cerebrovascular symptomatology have significantly improved responses for active compared to sham stimuli.

## Methods

### Data and Grouping

We adopted data from the study in Moussavi et al.^11^; out of the 135 participants who finished the study and their data were analyzed, 90 were randomly assigned to active rTMS treatment (either in 2 or 4 weeks of treatment) and 45 were assigned to sham rTMS treatment. Since the study results showed little difference between the sham and active groups post-treatment, in this study, we further investigate the responses of the patients with cerebral vascular symptomology (based on their modified Hachinski Ischemic Scores (HIS)^15,16^) as a measure of their impact on cognitive changes post-treatment.

A HIS score ⩾ 7 is argued to be representative of Vascular Dementia (VaD).^17^ A HIS score > 4 represents some vascular symptomology (eg, some of hypertension, cardiovascular disease, diabetes, or a history of stroke).^18^ A HIS score > 4 and <7 is claimed to be representative of MxD (mixed AD/VaD dementia).^17^ Alternative scales to diagnose VaD and “AD with cerebrovascular disease (cvd)” include the National Institute of Neurological Disorders and Stroke-Association Internationale pour la Recherche et l’Enseignement en Neurosciences (NINDS-AIREN) scale^19^ that has the advantage of including imaging data in their scoring, a limitation of the HIS. However, NINDS-AIREN lacks sensitivity as a neuropathological study demonstrated a sensitivity/specificity for possible VaD classification using the NINDS-AIREN scale of 55/84%.^19^ On the other hand, using an HIS threshold of 4 without imaging data 73.3% are correctly determined as AD and AD with vascular symptomatologies, and if an HIS threshold of 3 is used the accuracy increases to 77.8%.^17^ As stated by Knopman, 2001, “The *HIS*, while lacking neuroimaging criteria, may be more suitable for identifying the majority of dementia patients with vascular dementia (symptomatology), that is, those with at least some cerebrovascular pathology, because of the low sensitivity of the NINDS-AIREN and California criteria.^19^” It has become increasingly clear that the presence and extent of white matter hyperintensities (WMH), is often a radiographic marker of small cerebral vessel disease and an important predictor of the life-long risk of stroke, cognitive impairment,^20,21^ and functional disability.^22^

Thus, we made a modified HIS to be a 14-point scale by adding WMH as an extra 1 point to the original 13 points, similar to our previous studies.^23,24^ The WMH was identified from the brain’s MRI diagnostic report of each participant. Those with HIS ⩾ 4 were labeled ADcvd_4._ Those with a HIS < 4 were labeled AD_4_. To further examine the response efficacy’s sensitivity to cvd a second HIS threshold of 2 was also considered. If the HIS threshold of HIS ⩾ 2 was used, they were labeled or ADcvd_2_. Those with a HIS < 2 were labeled AD_2_. Tables 1 and 2 shows the patients’ demographics. AD and ADcvd subjects received active (N: ADcvd_4_active = 28, ADcvd_2_active = 53, AD_4_active = 65, AD_2_active = 37) or sham (ADcvd_4_sham = 12, ADcvd_2_sham = 28, AD_4_sham = 33, AD_2_sham = 17) stimuli.

**Table 1.** Average Sham group ADAScog scores and demographics. (AD_2/4_ is membership to both AD_2_ and AD_4_).

Group	Age	baseline	Week 5	Week 12	Week 20	Week 28	HIS	N	Gender ratio
AD_2/4_	70.59	23.71	22.28	23.47	23.67	23.49	0.53	17	0.47
AD_4_ OR ADcvd2	75.13	25.87	22.48	22.54	24.63	25.38	2.56	16	0.38
ADcvd_4_	80.58	20.11	16.95	17.86	19.06	20.33	5.00	12	0.42
ADcvd_2/4_	77.46	23.40	20.11	20.53	22.24	23.21	3.61	28	0.39

**Table 2.** Average active group demographics.

AD_2/4_	71.14	24.12	21.91	20.98	23.90	25.06	0.35	37	0.46
AD_4_ OR ADcvd2	75.50	24.41	22.88	23.01	25.11	26.70	2.39	28	0.50
ADcvd_4_	74.96	25.01	24.12	24.07	27.07	28.32	4.72	25	0.32
ADcvd_2/4_	75.25	24.69	23.47	23.51	26.03	27.47	3.49	53	0.42

### Statistical Analysis

The rTMS treatment study had 5 assessments: baseline (Week 0, immediately post-treatment at Week 5 and then on Weeks 12, 20, and 28 (6 months post-treatment). The averaged changes of ADAS-Cog scores of the weeks 5, 12, 20, and 28 with respect to baseline for each group are presented in Table 3.

**Table 3.** Descriptive statistics for CVD groups (HIS4).

	HIS4	Mean	Std. deviation	N
change @W5	ADcvd_4_active	–0.89	4.39	25
	ADcvd_4_sham	–3.17	6.68	12
	Total	–1.63	5.26	37
change @W12	ADcvd_4_active	–0.95	4.13	25
	ADcvd_4_sham	–2.25	6.13	12
	Total	–1.37	4.82	37
change @W20	ADcvd_4_active	2.05	5.62	25
	ADcvd_4_sham	–1.06	7.17	12
	Total	1.05	6.24	37
change @W28	ADcvd_4_active	3.31	5.82	25
	ADcvd_4_sham	0.22	5.00	12
	Total	2.31	5.69	37

A mixed model of repeated measure analysis of variance (ANOVA) was conducted between the ADcvd_*x*_ or AD_*x*_ groups in active and sham stimulation groups, where *x* represents either 2 or 4. Post-hoc analysis, simple model analysis, and *t*-tests are presented as required. We mainly investigated the following hypotheses:

1.  ADcvd_*x*_active and ADcvd_*x*_sham have significantly different ADAScog change results across time.
2.  The AD_*x*_active group’s ADAS-Cog changes will show significantly more improvement within 2 months of treatment (ie, Weeks 5 and 12) than the AD_*x*_sham group.

## Results

### Results of Investigating Hypothesis 1 (ADcvd_*x*_active vs ADcvd_*x*_sham) with HIS4 Threshold

**Figure 1.**
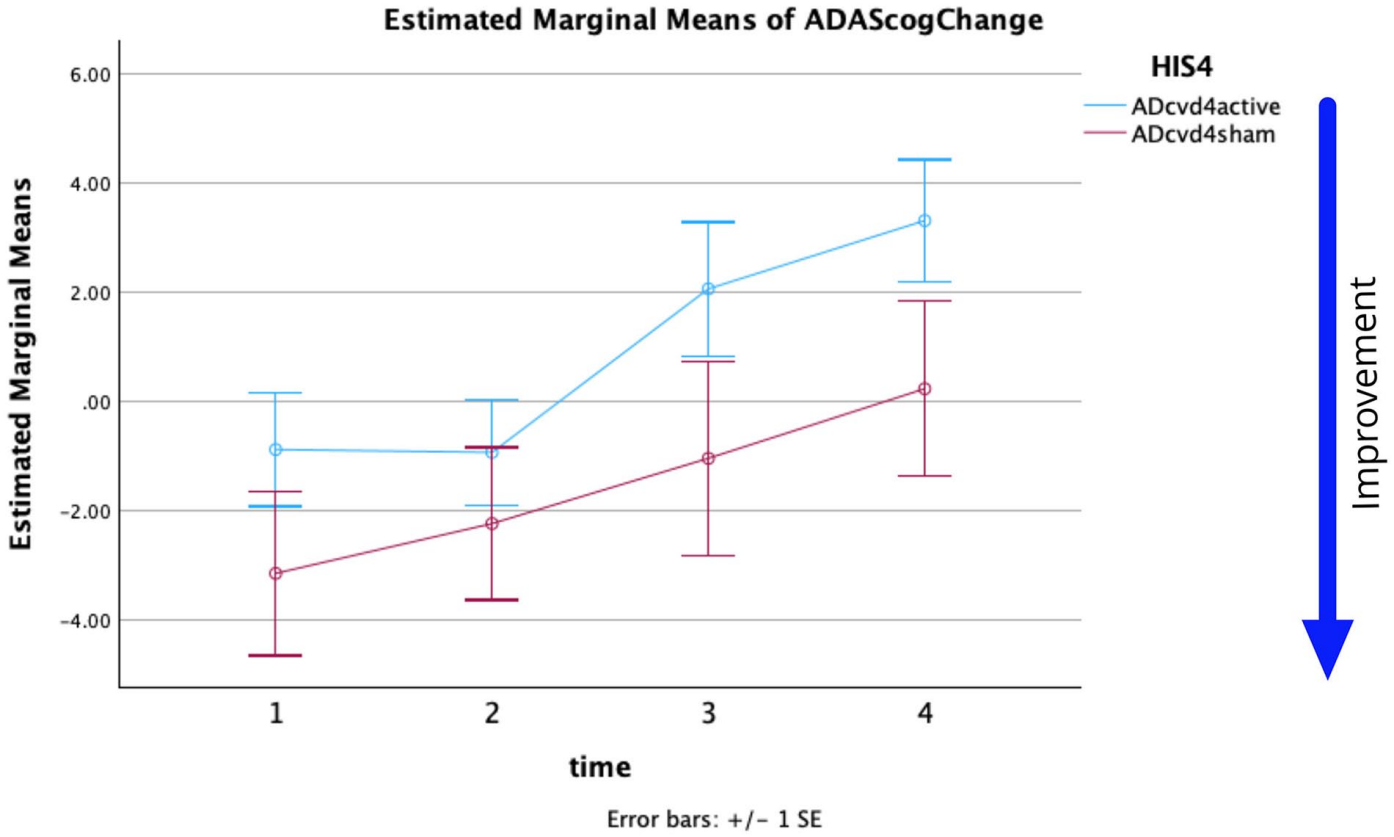
For HIS = 4 threshold estimated marginal means of ADAScog score change versus time. (Time 1, 2, 3, 4 = W5, W12, W20, W28).

Figure 1 shows the plots of the estimated marginal means of ADAS-Cog changes versus assessment times with respect to the baseline for ADcvd_4_active and ADcvd_4_sham (Tables 1 and 2). A repeated-measures ANOVA was performed to evaluate the effect of time on ADAS-Cog scores for these 2 groups. Mauchly’s test indicated that the assumption of sphericity had been violated, *χ*^2^(5) = 15.334, *p* = .009 and therefore the degrees of freedom were corrected using Greenhouse-Geisser estimates of sphericity (*ε* = 0.780). The effect of time on ADAS-Cog changes was significant *(F*(2.341, 81.930) = 8.368, *p* ⩽ .001, partial *η*^2^ = 0.193). The interaction of time * group was not significant *(F*(2.341, 81.930) = 0.477, *p* = .653, partial *η*^2^ = 0.013). The effect of the group on ADAS-Cog change was not significant (*F*(1,35) = 2.344, *p* = .135, partial η^2^ = 0.063).

Post-hoc pairwise comparisons with a Bonferroni adjustment indicated there was for ADcvd_4_active a significant difference between the measured ADAS-Cog change at times T1 to T3, T1 to T4, T2 to T4 (*p* = .035, .006, .008, respectively).

### Results of Investigating Hypothesis 1 (ADcvd_*x*_active vs ADcvd_*x*_sham) with HIS2 Threshold

**Figure 2.**
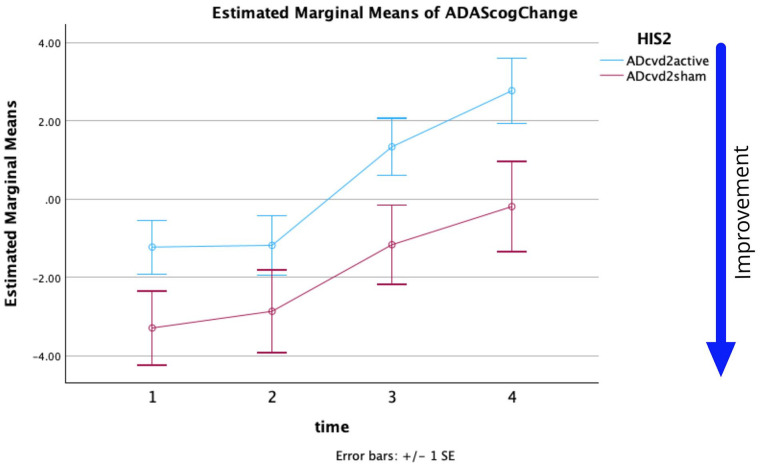
For HIS = 2 threshold estimated marginal means of ADAS-Cog score change versus time. (Time 1, 2, 3, 4 = W5, W12, W20, W28).

Figure 2 shows the plots of the estimated marginal means of ADAS-Cog changes versus assessment times with respect to the baseline for ADcvd_2_Active and ADcvd_2_Sham (Table 4). A repeated-measures ANOVA was performed to evaluate the effect of time on ADAS-Cog scores for these 2 groups. Mauchly’s test indicated the assumption of sphericity had been violated, *χ*^2^(5) = 12.991, *p* = .024; therefore, the degrees of freedom were corrected using Huynh-Feldt estimates of sphericity (*ε* = 0.950). The effect of time on ADAS-Cog change was significant *(F*(2.849, 225.081) = 15.787, *p* ⩽ .001, partial *η*^2^ = 0.167). The interaction of time × group was not significant (*F*(2.849, 225.081) = 0.409, *p* = .736, partial *η*^2^ = 0.005). The effect of group (ADcvd_4_active or ADcvd_4_sham) on ADAS-Cog change was significant (*F*(1,79) = 4.781, *p* = .032, partial *η*^2^ = 0.057). There were significant differences (*p* = .032) namely that ADcvd_2_sham when compared with ADcvd_2_active showed either more improvement or less decline.

**Table 4.** Descriptive Statistics for CVD groups (HIS2).

	HIS2	Mean	Std. deviation	N
change @W5	ADcvd_2_active	–1.23	4.78	53
	ADcvd_2_sham	–3.30	5.445	28
	Total	–1.94	5.09	81
change @W12	ADcvd_2_active	–1.18	5.07	53
	ADcvd_2_sham	–2.87	6.48	28
	Total	–1.77	5.61	81
change @W20	ADcvd_2_active	1.34	5.16	53
	ADcvd_2_sham	–1.17	5.67	28
	Total	0.47	5.44	81
change @W28	ADcvd_2_active	2.77	6.50	53
	ADcvd_2_sham	–.19	5.28	28
	Total	1.75	6.24	81

Post-hoc pairwise comparisons with a Bonferroni adjustment indicated that there was a significant difference for ADcvd_2_active between the ADAS-Cog change at times T1 to T3, T1 to T4, T2 to T3, T2 to T4 (*p* = .004, <.001, .009, <.001, respectively). Post-hoc pairwise comparisons with a Bonferroni adjustment indicated that there was a significant difference for ADcvd_2_sham between the ADAS-Cog change at times T1 to T3, T1 to T4 (*p* = .045, .015, respectively). A comparison of the simple effects of time within each group based on linearly independent pairwise comparison of estimated marginal means shows only the ADcvd_4_sham group had no significant main effect across time (Table 5).

**Table 5.** rmANOVA simple effects results. CVD groups.

Group	Degrees of freedom	*F*	*p*	η^2^	Power	Sphericity test
ADcvd_4_active	(3,96)	2.72	.049	0.08	0.64	
ADcvd_4_sham	(1.765,19.419)	1.94	.174	0.15	0.33	G-Geisser
ADcvd_2_active	(2.737,142.31)	13.84	<.001	0.21	1.0	Huynh-Feldt
ADcvd_2_sham	(3,81)	5.37	.002	0.17	0.92	

From the statistics and Figures 1 and 2 it can be seen:

1.  The effect of time on the ADAS-Cog change measure was significant for HIS2 and HIS4. Simple effects show only ADcvd_4_active had no simple effect across time but ADcvd_2_sham did (*p* = .002) supporting the hypothesis sham stimulation of AD_2_cvd patients had a significant measurable positive effect (which decreased over time).
2.  The noticeable positive effects of ADcvd_2_sham stimulation lasts at least until week 20 compared to week 12 for ADcvd_2_active stimulation.
3.  Post-hoc pairwise comparisons with a Bonferroni adjustment indicated that there were significant differences between the ADcvd_2_active and ADcvd_2_sham groups (*p* = .032) namely ADcvd_2_sham when compared with ADcvd_2_active showing either more improvement or less decline.

The ADcvd_4_sham group (Figure 1 red curve) noticeably shows more improvement or less decline than the ADcvd_4_active group (blue curve). However, note that an ADAS-Cog score improvement ⩾ 3 is considered significant.^25^ Post-hoc pairwise comparisons with a Bonferroni adjustment indicated that there was no significant difference between the ADcvd_4_active and ADcvd_4_sham groups, (*p* = .135). Furthermore, for ADcvd_4_sham a potential (non-significant) improvement persisted up to Week 20 post baseline, while the ADcvd_4_active group appeared to show little or no improvement after Week 12.

To further increase our understanding of the degree of cerebrovascular symptomology on the rTMS efficacy, we also looked at grouping the participants with an HIS2 threshold as described in the Methods. Figure 2 is similar to Figure 1 but for the HIS2 thresholding. There were significant differences between the ADcvd_2_active and ADcvd_2_sham groups (*p* = .032) namely ADcvd_2_sham when compared with ADcvd_2_active showing either more improvement or less decline. Similar to the HIS4 potential trend, for ADcvd_2_sham the improvement persisted at least up to Week 20, while the ADcvd_2_active group showed no improvement after Week 12.

### Results of Investigating Hypothesis 2 (AD_*x*_active vs AD_*x*_sham Improvements at Weeks 5 and 12) with for His4

As for the second hypothesis, we questioned if there was a significant improvement (one sided question, *p* = .1) in cognition in the week 5 or week 12 ADAS-Cog change (Bonferroni corrected *p* = .025) for AD_*x*_active compared to AD_*x*_sham stimulation.

For HIS4 thresholding, a repeated-measures ANOVA was performed to evaluate the effect of time on the ADAS-Cog changes. The means and standard deviations for ADAS-Cog change are presented in Table 6.

**Table 6.** Descriptive Statistics. AD groups, HIS4.

	HIS4	Mean	Std. deviation	N
change @W5	AD_4_active	–1.91	4.67	65
	AD_4_sham	–2.38	4.76	33
	Total	–2.07	4.68	98
change @W12	AD_4_active	–2.38	5.19	65
	AD_4_sham	–1.74	5.89	33
	Total	–2.17	5.41	98

Mauchly’s test indicated that the assumption of sphericity had not been violated, *χ*^2^(5) = 1.0. The effect of time on ADAS = Cog change was not significant at the .05 level, (*F*(1,96) = 0.033, *p* = .856, partial η^2^ = 0.0). The interaction of time * group was not significant at the .05 level (*F*(1,96) = 1.394, *p* = .241, partial η^2^ = 0.014). The effect of the group on ADAS-Cog changes was not significant at the .05 level, (*F*(1,96) = 0.008, *p* = .928, partial η^2^ = 0.0).

### Results of Investigating Hypothesis 2 (AD_*x*_active vs AD_*x*_sham Improvements at Weeks 5 and 12) with for His2

For HIS2 thresholding, a repeated-measures ANOVA was performed to evaluate the effect of time on the ADAS-Cog changes. The means and standard deviations for ADAS-Cog changes are presented in Table 7.

**Table 7.** Descriptive Statistics. AD groups, HIS2.

	HIS2	Mean	Std. deviation	N
change @5	AD_2_active	–2.21	4.30	37
	AD_2_sham	–1.43	4.89	17
	Total	–1.96	4.46	54
change @12	AD_2_active	–3.14	4.57	37
	AD_2_sham	–.24	4.49	17
	Total	–2.22	4.70	54

Mauchly’s test indicated that the assumption of sphericity had not been violated, *χ*^2^(5) = 1.0. The effect of time on the ADAS-Cog changes was not significant at the .05 level, (*F*(1,52) = 0.049, *p* = .825, partial *η*^2^ = 0.001). The interaction of time × group was not significant at the .05 level (*F*(1,52) = 3.142, *p* = .082, partial *η*^2^ = 0.057). The effect of the stimulation applied does not depend on time. The effect of group (AD_4_active or AD_4_sham) on ADAS-Cog changes was not significant at the .05 level (*F*(1,52) = 2.426, *p* = .125, partial *η*^2^ = 0.045). The lack of almost parallel traces in Figure 4 supports a simple effects analysis.

Pairwise comparisons of group × time showed, only at time 2, a significant (*p* = .05) mean difference (Mean difference = −2.899, std. error = 1.331, sig = 0.034, Bonferroni corrected). The same result was obtained from pairwise comparisons using a Univariate ANOVA at time T2 (*F*(1,52) = 4.746, sig = 0.034, η^2^ = 0.084, power = 0.571). The p-value remains at .05 as there are 2 time comparisons and the test is one sided making the result significant for the hypothesis question “did AD_2_active produce an improvement in cognition compared to AD_2_sham.” Comparatively, an independent samples *t*-test at T2 gave for a one-sided test with Bonferroni correction and unequal variances t = 2.193, *p* = .036 (0.018 × 2), Cohen’s *d* = −0.638. At T1 there was no significant difference. The mean ADAS-Cog change score was affected by the group with a medium effect size.

**Figure 3.**
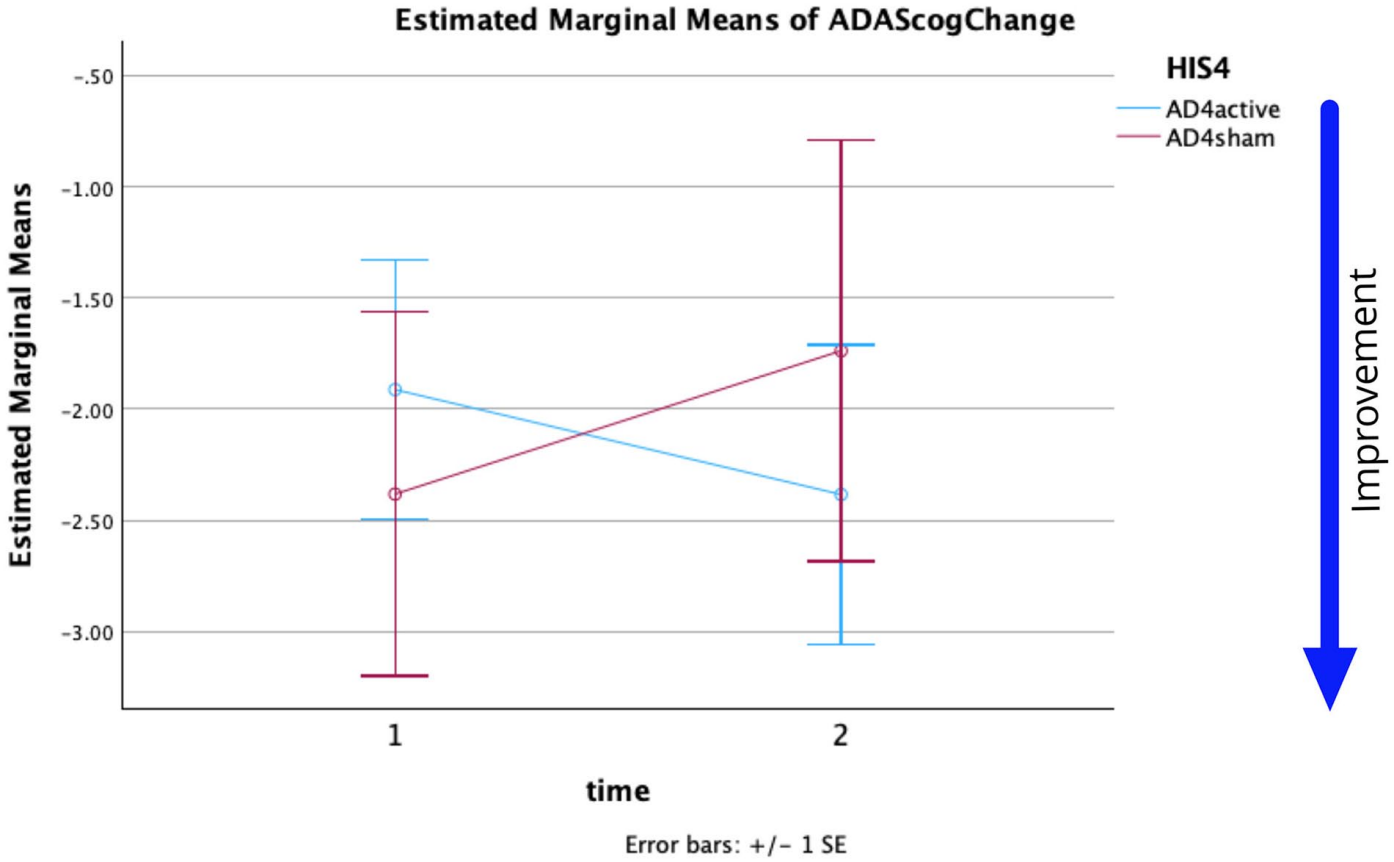
For HIS = 4 threshold estimated marginal means of ADAS-Cog changes versus time. (Time 1, 2 = W5, W12).

**Figure 4.**
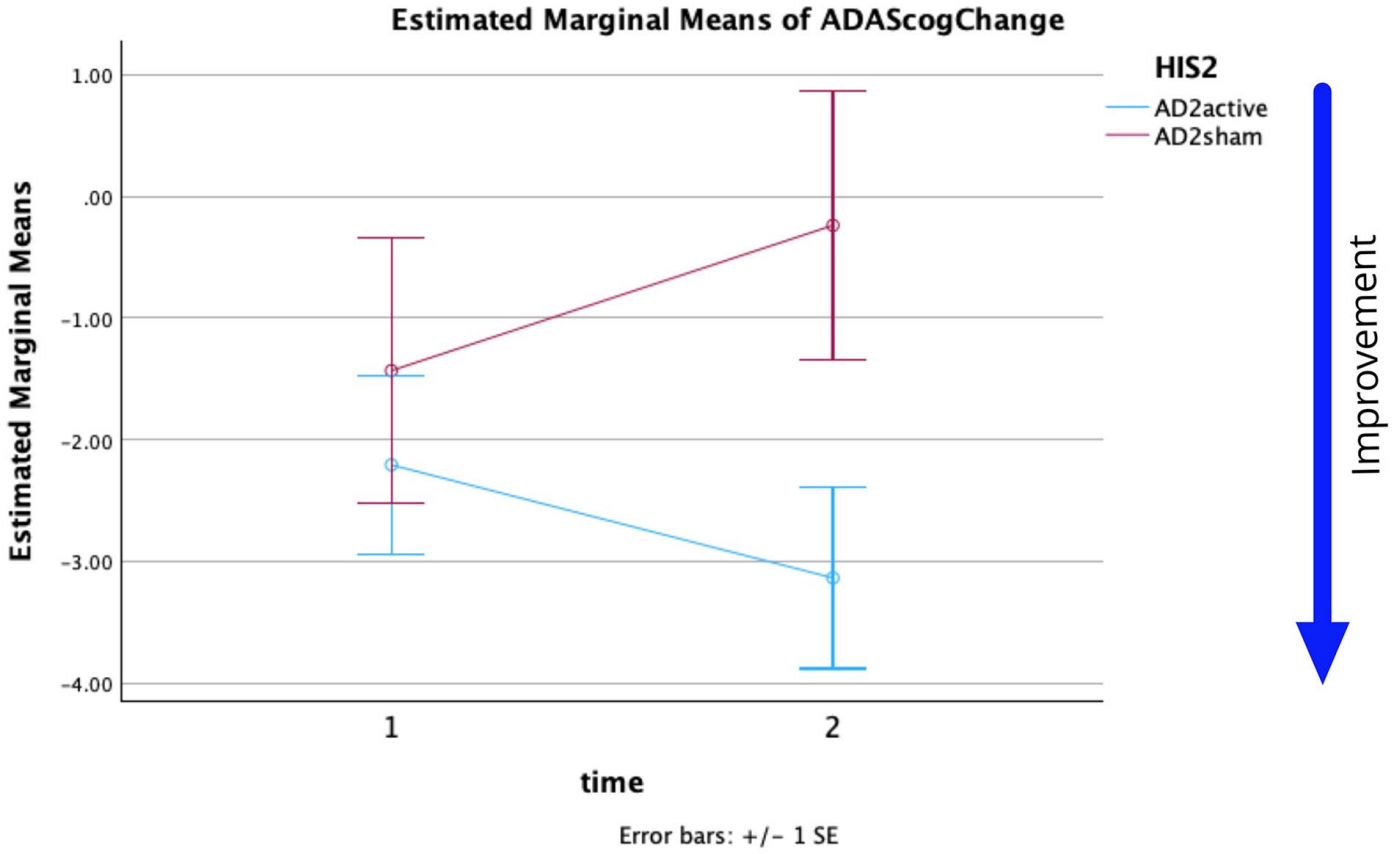
For HIS = 2 threshold estimated marginal means of ADAS-Cog changes versus time. (Time 1, 2 = W5, W12).

From the statistics and Figures 3 and 4 it can be seen:

1.  AD_2_active was more effective than AD_2_sham at time T2.

## Discussion

The largest rTMS treatment study for AD,^11^ wherein we adopted their data for this current study, showed no significant differences between the sham and active groups either immediately post-treatment or at follow-up assessments up to 6 months. We hypothesized and showed that the sham rTMS stimulus can evoke a measurable response and is more effective on those with ADcvd, and that that could be supportive of a vascular mechanism likely linked to the shallower sham stimulus penetration. In other words, the level of cerebrovascular disease may be an impacting factor on rTMS efficacy, as implied by a recent study.^12^ There are 3 main outcomes of this study: (1) the sham coil used outputs a neuromodulator stimulus capable of impacting, in particular, ADcvd_2_ patients; (2) ADcvd patients likely need to be treated as a separate population when it comes to rTMS stimulation; and (3) a stimulus akin to the sham stimulus applied herein might be a suitable or even better stimulus therapy for ADcvd patients.

As mentioned in the introduction other studies support the neuromodulator effects of the applied sham coil stimulation.^2,3,11^ Statistical data and Figures 1 and 2 show there are significant differences between active and sham stimulation for ADcvd_2_. We can only speculate as to why improvements to the ADcvd_2_ sham group improvements appear to persist longer. One future hypothesis worth exploring may be long term changes occur in neuro-vascular coupling and, in particular, capillary pericyte resting states.^26^

As for why the responses of the sham group of the rTMS treatment study^11^ were similar to the responses of the active groups across the time, those authors argued a sham coil with its weaker magnetic coil possibly induces a weak current in the brain that while it is not strong enough to evoke a direct neural response, it may act like the way a weak transcranial alternating stimulation (eg, tACS) works.^27^ While rTMS directly increases cerebral blood flow in the targeted brain region by stimulating neuronal activity,^28^ tACS indirectly increases blood flow by modulating brain oscillation and neuronal synchronization leading to improved metabolic demand and localized increases in cerebral blood flow.^29^ tACS has also shown the potential to modulate brain activity and enhance cognitive function.^27^ It modulates brain oscillatory networks by applying alternating currents, which can synchronize neuronal activities and potentially boost cognitive processes related to memory and attention.^27^ tACS at Gamma-band frequencies (30-100 Hz) have been specifically linked to working memory and higher cognitive functions, respectively.^30^

Investigating the potential similarity of the sham coil and tACS effects on the brain function is beyond the scope of this paper as that requires derivation of the induced electrical fields in the brain by simulation for these 2 modalities. However, in this paper, we focused on whether cvd plays a role in the response of patients to rTMS in the short and long-term.

Recently, tACS and transcranial direct current stimulation (tDCS) have been applied as a therapeutic stimulus for AD and ADcvd patients for example, Moussavi et al.^29^ Cammisuli et al.,^31^ and Herrmann et al.^32^ Both of these stimulus types purportedly act closer to the skull surface and can be linked to vascular and/or metabolic processes and may be more comparable with the sham stimulus applied herein.^33,34^ The ADcvd patients, in particular, may be benefiting from a sham stimulus capable of improving either or both of damaged vascular flow and related metabolism. If the vascular flow or metabolism is relatively healthy, the sham stimulus would likely have a much smaller impact as observed in AD participants (Figure 4).

It has also been shown in other studies that the measured physiological characteristics of AD and ADcvd populations can be quite different. For example, in Lithgow et al.^24^ and Dastgheib et al.^35^ it has been shown, using Electrovestibulography, the recorded field potentials and low frequency interval histograms (IH_33_) from AD and ADcvd patients can be used to discriminate between them.

The results of the mixed effect model for each thresholding (HIS4 or HIS2) show ADAS-Cog changes significantly differed across weeks and also across the HIS2 groups. Limitations on this study’s analysis include unbalanced groupings (approximately 2.3:1 for HIS4 and 2:1 for HIS2 active:sham respectively) and limited sample size in some groups (12 in ADcvd_4_sham). Despite these limitations the repeated measures ANOVA analysis generated a *p* = .032 for analysis of Groups when using HIS2 thresholding. The cvd data in Figures 1 and 2 support sham rTMS having a positive outcome at least to W12 (2 months post-treatment). Subsequent measurements were significantly different and showed a decline. Data support assertions that ADcvd_2_sham stimulation was more effective than ADcvd_2_active stimulation, the improvements persisting beyond W20. The limited sample size remains this study’s biggest limitation despite this being the largest rTMS longitudinal study conducted so far. Lastly, the exclusion of cvd patients from Figure 4 showed AD_2_active compared to AD_2_sham can produce more improvement at week 12 and this is supportive of rTMS treatments for AD patients.

Given the results of this study and sham coil’s benefits for ADcvd groups, future studies could investigate the efficacy of active rTMS at lower intensities such as 25% of the resting motor threshold to be comparable with sham coil’s intensity but also over a larger area of the brain; perhaps the new coil such as the Brainsway rotating field coils^36^ could be better to be used for future investigations.

## Conclusions

In our dataset, the rTMS “SHAM” stimulus did evoke a measurable response. This was more effective on ADcvd_2_sham than the ADcvd_2_active groups. This is supportive of a vascular mechanism likely linked to the depth-wise shallower stimulus penetration of the sham coil. In addition, by excluding ADcvd_2_ participants, the remaining AD participants in the active group showed significant improvement compared to those in the sham group and this lasts up to 2 months post-treatment.

## Statements and Declarations

**Data Availability:** The datasets generated during and/or analyzed during the current study are available from the corresponding author on reasonable request.
